# Single cell transcriptomics of neighboring hyphae of *Aspergillus niger*

**DOI:** 10.1186/gb-2011-12-8-r71

**Published:** 2011-08-04

**Authors:** Charissa de Bekker, Oskar Bruning, Martijs J Jonker, Timo M Breit, Han AB Wösten

**Affiliations:** 1 Microbiology and Kluyver Centre for Genomics of Industrial Fermentations, Institute of Biomembranes, Utrecht University, Padualaan 8, 3584 CH Utrecht, The Netherlands; 2 Microarray Department and Integrative Bioinformatics Unit, Swammerdam Institute for Life Sciences, University of Amsterdam, Science Park 904, 1098 XH Amsterdam, The Netherlands; 3 Netherlands Bioinformatics Centre (NBIC), Geert Grooteplein 28, 6525 GA Nijmegen Nijmegen, the Netherlands

## Abstract

Single cell profiling was performed to assess differences in RNA accumulation in neighboring hyphae of the fungus *Aspergillus niger*. A protocol was developed to isolate and amplify RNA from single hyphae or parts thereof. Microarray analysis resulted in a present call for 4 to 7% of the *A. niger *genes, of which 12% showed heterogeneous RNA levels. These genes belonged to a wide range of gene categories.

## Background

Cellular heterogeneity within an isogenic cell population is a widespread event in both prokaryotic and eukaryotic organisms. Heterogeneity of cells can be beneficial for the organism in many ways. Many documented cases of phenotypic variability in microorganisms relate to responses to environmental stress. This suggests that phenotypic variation aids in the survival of cells under adverse conditions and therefore may be an evolvable trait [1,2]. It has been shown that mycelia of filamentous fungi are also heterogeneous. For instance, protein secretion [3-5] and gene expression [6-9] are heterogeneous between zones of fungal colonies. These differences were explained by the availability of carbon source and by spatial and temporal differentiation [7]. Heterogeneous gene expression can even be found within a zone of a colony. In fact, expression of the glucoamylase gene *glaA*, the acid amylase gene *aamA*, the α-glucuronidase gene *aguA*, and the feruloyl esterase gene *faeA *is heterogeneous between neighboring hyphae at the periphery of the colony of *Aspergillus niger *[10,11]. Co-expression studies showed that hyphae that highly express one of these genes also highly express the other genes encoding secreted proteins [11]. Moreover, these hyphae highly express the glyceraldehyde-3-phosphate dehydrogenase gene *gpdA*, and are characterized by a high *18S *rRNA content. Taken together, it was concluded that at least two subpopulations of hyphae exist within the outer zone of the mycelium of *A. niger*. These subpopulations are characterized by a high and a low transcriptional activity, respectively [11]. The data implied also that the translational activity may be different in the two populations of hyphae.

Transcriptome analysis of single cells is an important tool to understand the extent of cellular heterogeneity and its underlying mechanisms. So far, whole genome expression analysis has been reported of an individual neuron and a single blastomere [12,13]. Here, we performed for the first time a single cell transcriptome analysis in a microbe. It is shown that the RNA composition of neighboring hyphae at the periphery of an *A. niger *mycelium is heterogeneous. Heterogeneity can be found in all functional gene classes (FunCats) as well as in rRNAs and tRNAs.

## Results

### Hyphal architecture at the periphery of a sandwiched colony

**Figure 1:**
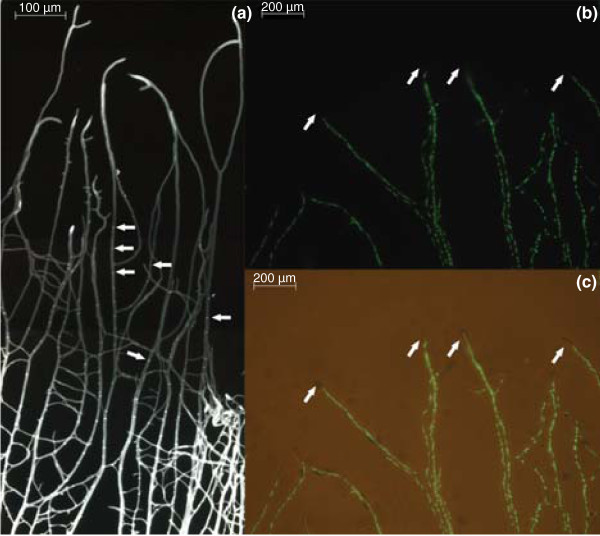
**Distribution of septa and nuclei in hyphae at the outer part of a sandwiched colony**. **(a) **Calcofluor white staining visualizing the septa within the hyphae (indicated by arrows). The first septum is positioned 400 μm from the apex of the hyphae. **(b) **GFP fused to H2B visualizing the nuclei. **(c) **Overlay of (b) with a bright field image. The region 10 to 20 μm from the apex is free from nuclei (indicated by arrows).

Distribution of nuclei and septa was monitored at the periphery of 7-day-old sandwiched colonies of *A. niger *using a fusion of the histone H2B protein and green fluorescent protein (H2B-GFP fusion) and calcofluor white, respectively. Septa were not detected within the first 400 μm from the tip (Figure 1a). After the first septum, septa were separated by 50 to 100 μm. Nuclei were found throughout the hypha, except for the region 10 to 20 μm from the tip (Figure 1b, c). Taken together, only part of the first compartment of hyphae of *A. niger *is analyzed when tip regions of 100 to 200 μm are dissected for RNA analysis (see below).

### RNA profiling of single hyphal tips

A reproducible RNA extraction and amplification protocol was developed to enable analysis of transcript profiles of selected (parts of) hyphae within a mycelium (Additional file 1). This protocol includes growth conditions and sample preparation, laser dissection, RNA isolation, and cDNA amplification and labeling. The protocol was used to isolate RNA from 1,000 hyphal tips (with a width of 3 to 4 μm and a length of 100 μm) from the outer periphery of 7-day-old sandwiched colonies of *A. niger *strain AR9#2. The RNA was spotted onto a nylon membrane and hybridized with an *18S *rDNA probe. The hybridization signal was compared to that of samples with a known RNA concentration. From this it was concluded that 1,000 hyphal tips with a length of 100 μm contain 1 ng of RNA (Additional file 2).

**Figure 2:**
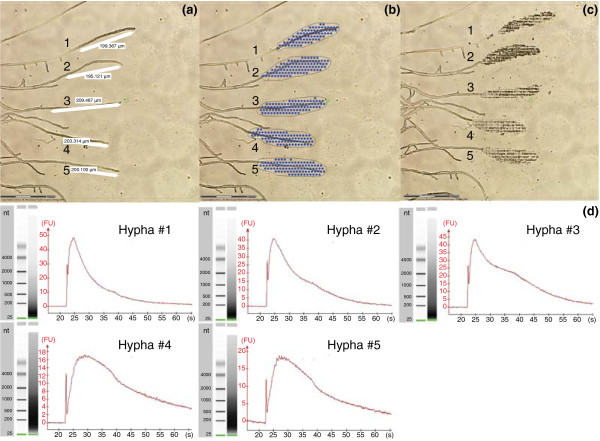
**Amplification of cDNA from the tips of five single neighboring hyphae**. **(a-c) **Apical regions of 200 μm of hyphae at the periphery of sandwiched colonies were selected by using the measuring (a) and drawing (b) tool. Fragments of a single hypha were catapulted into a cap of an Eppendorf tube by using the autoLPC option (c). RNA of the single hyphae was isolated, converted into cDNA and amplified. **(d) **The amplified cDNA was analyzed with a Bioanalyser. The electrophoresis gel image (nt, nucleotides; L, ladder) and the electropherogram (y-axis represents fluorescence units (FU); x-axis represents run time in seconds (s)) are given for the five samples. Amplicons of hyphae 1 to 3 and hyphae 4 and 5 were mainly 50 to 100 bp and 100 to 300 bp in length, respectively.

RNA was isolated from five single tips with a length of 200 μm of neighboring hyphae from the outermost region of a 7-day-old *A. niger *sandwiched colony. To this end, fragments of each hypha were catapulted into a cap of an Eppendorf tube using the autoLPC option (Figure 2a-c). After RNA isolation, half of the total RNA contained in each of the five samples was converted into cDNA. This cDNA was amplified to 5.9 to 10.1 μg with the WT-Ovation One-Direct RNA Amplification System (Nugen, San Carlos, CA, USA) and used for quantitative PCR (QPCR) and hybridization of Affymetrix *A. niger *gene chips (Affymetrix, Santa Clara, CA, USA). The amplicons of three of the samples (hyphae 1 to 3) were mainly 50 to 100 bp in length, while most of the amplicons of the other two hyphae (hyphae 4 and 5) had a length of 100 to 300 bp (Figure 2d). Notably, the latter two samples had been amplified on a different day than the former three samples.

### Hyphal heterogeneity analyzed by QPCR

The amplified cDNA samples of the five single hyphae were analyzed by QPCR. As a control, amplified cDNA was used from three biological replicates of a pool of 100 hyphal tips and of mycelium of the whole periphery of sandwiched colonies. Cycle threshold (Ct) levels were determined for *18S *rRNA, *actin*, and *glaA *using 1 ng cDNA and six technical replicates for each sample. Ct values for the RNA samples from the periphery of the colony were very similar (Table 1). An F-test showed that the standard deviation of the biological replicates was not significantly higher than the maximum standard deviation obtained for one of the series of technical triplicates (*P *≤ 0.01; Additional file 3). Standard deviations found for the Ct values of the samples of 100 hyphal tips were between 1.5 and 8.4 fold higher when compared to the cDNA from the whole periphery but they were not significantly different from the technical replicates (*P *≤ 0.01; Table 1; Additional file 3). The differences in RNA levels were even more pronounced when individual hyphae were compared (Table 1; Additional file 3). The standard deviations for the Ct values of the *glaA *and *actin *genes of hyphae 1 to 5 were significantly higher when compared to the maximum standard deviation obtained for the technical replicates within this sample type. This difference was not observed with the *18S rRNA *gene. Similar results were obtained with the standard deviation of the levels of *18S rRNA*, and *glaA *and *actin *mRNA when hyphae 1 to 3 and hyphae 4 and 5 were analyzed separately (Additional file 3). This shows that the differences in RNA levels are not due to a batch effect.

**Table 1:** Accumulation of RNA is heterogeneous between hyphae at the periphery of an A.*niger colony*

Gene	Sample type	μCt	σ	σ technical
*18S*	1 hypha	20.23	2.40	0.53-1.26
	100 hyphae	17.39	0.42	0.26-0.43
	5 pg periphery	12.58	0.28	0.15-0.35
*actin*	1 hypha	30.30	6.07	0.11-1.03
	100 hyphae	28.15	4.80	0.12-1.71
	5 pg periphery	18.05	0.57	0.09-0.22
*glaA*	1 hypha	25.47	4.63	0.06-0.66
	100 hyphae	24.51	2.22	0.10-0.22
	5 pg periphery	18.65	0.49	0.10-0.14

QPCR was performed using 1 ng of cDNA amplified from RNA of tips of single neighboring hyphae from the outer periphery of a sandwiched colony, from RNA of a pool of 100 of such tips, and from RNA of the 3-mm-wide periphery of sandwiched colonies of *A. niger*. The average cycle threshold (μCt) and their standard deviations (σ) are given for *18S *rDNA and the *actin *and *glaA *genes. σ technical represents the range in standard deviations obtained for the six technical replicates for each of the biological replicates.

### Hyphal heterogeneity analyzed by microarrays

Biotin-labeled amplified cDNA of the single hyphal tips was hybridized to Affymetrix *A. niger *gene chips. Based on MAS5.0 detection calls, transcripts of 4.1 to 6.7% of the genes had a present call in each of the single hyphae (Additional file 4). Genes with an absent call had generally low signal values in hybridization experiments where 500 pg RNA from the periphery or from a pool of 500 hyphal tips was used [14] (Additional file 5). The scale factors of the sample types had a difference < 5-fold. Due to the low number of present calls, this difference in scale factors was considered to be low enough to normalize and analyze the samples as a whole. In total, 2,608 of the 14,455 probe sets had a present call in at least one of the samples of the single hyphae (Additional file 6). These probe sets were found to comprise all 19 different class I functional categories (FunCats) as well as the non-FunCat categories tRNA and rRNA. Almost half of the detectable probe sets belonged to unclassified proteins (Table 2). Metabolism was the second largest group, with 550 hybridizing probe sets. Categories with more than 50 probe sets with a present call comprised protein fate (148), transcription (116), cell cycle and DNA processing (106), cellular transport and transport mechanisms (76), protein synthesis (75), and cell rescue, defense and virulence (52). The categories with a lower number of present calls generally comprised a small number of total probe sets. In most categories 10 to 30% of the probe sets had a present call. This was 50 to 75% for the categories rRNA, tissue localization, and protein with binding function or co-factor requirement. Similar results for the FunCat analysis were obtained when hyphae 1 to 3 and hyphae 4 and 5 were analyzed separately (data not shown). This shows that the analysis was hardly, if at all, affected by a batch effect.

**Table 2:** Classification of probe sets with a present call in at least one of the five arrays of a single hypha

Category	Number of probe sets with a present call	Total number of probe sets	Percentage of probe sets present
01 Metabolism	550	3023	18.2
02 Energy	25	117	21.4
03 Cell cycle and dna processing	106	476	22.3
04 Transcription	116	715	16.2
05 Protein synthesis	75	241	31.1
06 Protein fate (folding, modification, destination)	148	616	24
08 Cellular transport and transport mechanisms	76	438	17.4
10 Cellular communication or signal transduction mechanism	31	179	17.3
11 Cell rescue, defense and virulence	52	267	19.5
13 Regulation of or interaction with cellular environment	8	73	11
14 Cell fate	15	104	14.4
25 Development (systemic)	6	26	23.1
29 Transposable elements, viral and plasmid proteins	12	62	19.4
30 Control of cellular organization	5	29	17.2
40 Sub-cellular localization	19	137	13.9
45 Tissue localization	1	2	50
63 Protein with binding function or co-factor requirement	1	2	50
67 Transport facilitation	5	44	11.4
99 Unclassified proteins	1,279	7,670	16.7
rRNA	6	8	75
tRNA	39	144	27.1

Classification of the 2,608 probe sets is based on class I FunCats as well as the non-FunCat categories tRNA and rRNA.

**Figure 3:**
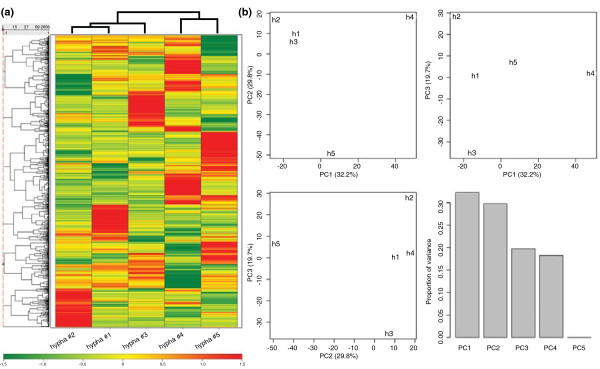
**Hierarchal clustering and principal component analysis of RNA profiles of five neighboring hyphae**. **(a, b) **Hierarchal clustering (a) and principal component analysis (PCA) (b) were done on the basis of the 2,608 probe sets that had a present call in at least one of the single hyphae. Clustering was done using complete linkage as clustering method with 1-correlation as distance measure. The Z-scores of the log2 RMA signal values of the 2,608 probe sets were used for clustering and PCA.

Hierarchical clustering of the hyphae was done on basis of the Z-scores of the log2 signals of the robust multi-array analysis (RMA) using the 2,608 probe sets that had a present call in at least one of the hyphae (Figure 3a). As a distance (d) measure 1-the Pearson correlation was used (a distance of 0 means that the samples are identical; a distance of 2 means the samples are completely different). Hyphae 1 and 2 were least distant (d = 1.10). The correlation between hypha 3 and hyphae 1 and 2 and between hyphae 4 and 5 was similar (d = 1.22 and d = 1.24, respectively). Principal component analysis (PCA) revealed that hypha 4 was separated from the other samples in the first principal component. Hypha 5 was separated in the second principal component, whereas hyphae 2 and 3 were separated in the third principal component (Figure 3b). In the next analysis, overrepresentation of functional gene categories was tested for all probe sets with a present call in each of the individual hyphae. This revealed that the non-FunCat categories rRNA and tRNA were overrepresented in all five hyphae (Table 3; Additional file 7). Ribosome biogenesis was overrepresented in four hyphae, whereas other export and secretion systems and proteolytic degradation were overrepresented in two of the hyphae. Similar results for the over-representation analysis were obtained when hyphae 1 to 3 and hyphae 4 and 5 were analyzed separately (data not shown). This shows that the analysis was hardly, if at all, affected by a batch effect.

**Table 3:** Overrepresented functional gene categories within the set of genes with a present call in a single hypha

	Number of probe sets	Hypha number^a^				
		
Functional gene category		1	2	3	4	5
03.01.03 DNA synthesis and replication	96	1	0	0	0	0
03.01 DNA processing	240	1	0	0	0	0
05.01 Ribosome biogenesis	138	1	1	1	0	1
06.13.04 Lysosomal and vacuolar degradation	32	0	0	1	0	0
06.13 Proteolytic degradation	198	0	1	1	0	0
08.16.99 Other export and secretion systems	15	0	0	1	1	0
29.01 LTR retro-elements (retro-viral)	28	0	0	1	0	0
40.05 Centrosome	9	0	0	0	1	0
67.04.01.02 Other cation transporters (NA, K, CA, NH4, etc.)	33	0	0	0	1	0
rRNA	8	1	1	1	1	1
tRNA	144	1	1	1	1	1

^a^1, overrepresentation of a category (*P *< 0.01); 0, absence of overrepresentation (*P *> 0.01). For *P*-values see Additional file 7.

**Figure 4:**
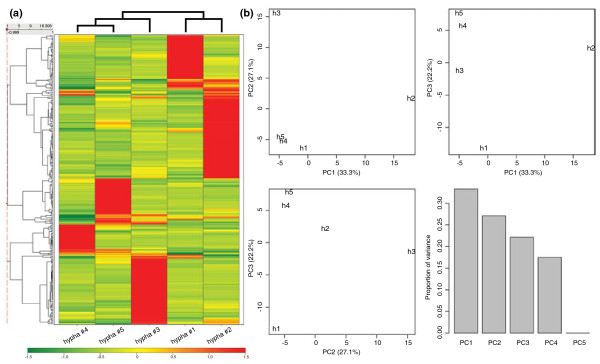
**Hierarchal clustering and PCA of RNA profiles of five neighboring hyphae**. **(a, b) **Hierarchal clustering (a) and PCA (b) were done on the basis of the 308 probe sets that had a present call in at least one out of five hyphae and that showed a standard deviation > 0.5 between the signal values. Clustering was done using complete linkage as clustering method with 1-correlation as distance measure. The Z-scores of the log2 RMA signal values of the 308 probe sets were used for clustering and PCA.

Within the 2,608 probe sets with a present call in at least one of the five single hyphae, 308 showed a relatively high standard deviation (> 0.5) between the log2 RMA signal values (Additional file 8). Within these probe sets, 5 out of 19 class I FunCats are not represented. These five categories (regulation of or interaction with cellular environment, development, tissue localization, protein with binding function or co-factor requirement, and transport facilitation) have relatively few members (with a maximum of 73 members). The hyphae were clustered based on the Z-scores of the signals of the 308 probe sets (Figure 4a). This revealed that hyphae 4 and 5 were most similar. Hypha 3 was more similar to hyphae 4 and 5 than to hyphae 1 and 2. PCA (Figure 4b) revealed that in the first principal component, hypha 2 separated from the other samples, whereas hypha 3 and hypha 1 were separated in the second and third principal components, respectively. Each hypha showed a cluster of genes with higher signals when compared to the other four hyphae (Figure 4a). These clusters were analyzed for overrepresentation of functional gene categories (Table 4; Additional file 7). This revealed that the classes ribosome biogenesis and tRNA were overrepresented in three of the five hyphae. The cluster of hypha 5 was not enriched in any functional category. In contrast, seven FunCats were overrepresented in the cluster of hypha 2, among which two are involved in energy. Separate analysis of hyphae 1 to 3 and hyphae 4 and 5 had an effect on the over-representation analysis. As mentioned above, the analysis was based on a list of highly variable genes with a present call in at least one sample. The results of the analysis were not different because of a batch effect but simply because it was based on three (or two) instead of five samples. Indeed, very similar significance values were obtained for almost all functional gene categories in Table 4 when the five hyphae were analyzed together or when hyphae 1 to 3 and 4 and 5 were analyzed separately (Additional file 9).

**Table 4:** Overrepresented functional gene categories within the set of genes that have a signal value with a standard deviation of > 0.5 between the five single hyphae

	Number of probe sets	Hypha number ^a^				
		
Functional gene category		1	2	3	4	5
02.13.03 Aerobic respiration	62	0	1	0	0	0
02.13 Respiration	93	0	1	0	0	0
02.19 Metabolsim of energy reserves (for example, glycogen, trehalose)	36	1	0	0	0	0
04.05.01.01 General transcription activities	107	0	1	0	0	0
05.01 Ribosome biogenesis	138	1	1	1	0	0
06.07.99 Other protein modifications	54	0	0	0	1	0
06.13 Proteolytic degradation	198	0	1	0	0	0
30.10 Nucleus	55	0	1	0	0	0
67.15 Electron or hydrogen carrier	48	0	1	0	0	0
tRNA	144	1	0	1	1	0

^a^1, overrepresentation of a category (*P *< 0.01); 0, absence of overrepresentation (*P *> 0.01). For *P*-values see Additional file 7.

The top 100 genes with the highest hybridization signal in each of the hyphae was selected (Additional file 10). This selection comprised a total of 207 genes. Of these genes, 43 and 18 are found within the top 100 of all 5 hyphae and of 4 hyphae, respectively (Additional file 11). A major part of these genes (19 out of 43 and 11 out of 18) encode unidentified proteins. Examples of genes that have a predicted function are the 4 rRNAs, 4 tRNAs and 14 genes involved in metabolism. A number of 119 genes (of which 43 encode unidentified proteins) were found in only one of the hyphae (Additional file 12). The glucoamylase gene *glaA*, the cellulase gene *eglb*, 7 genes encoding cytoplasmic ribosomal proteins and 13 tRNAs were among these genes. The 119 genes were randomly distributed in the top 100 of the different hyphae.

## Discussion

A disconnection has been observed between the average gene expression of a culture of isogenic cells and the gene expression of a single cell within such a culture [15,16]. Therefore, single cell analysis with high spatiotemporal resolution is needed to give an accurate understanding of the processes within a cell. Single cell analysis is not yet widely applied mainly because of the fact that the technologies that are needed are not fully developed. The fact that microbial cells are much smaller than those of plants and animals complicates the use of these technologies. Here, we developed a protocol for single cell transcriptome analysis of hyphae of the microbe *A. niger*. Using this protocol, it is shown that neighboring hyphae have a heterogeneous RNA composition despite the fact that they experience identical environmental conditions. Differences in RNA accumulation were shown to occur in all functional gene groups (FunCats) as well as in rRNAs and tRNAs. The observed fluctuations in RNA accumulation between individual hyphae are supported by GFP reporter studies and *in situ *hybridizations [10,11,17]. The variation in transcriptome composition is not the result of differences in growth rate. The hyphae that were selected had a similar diameter and extension rate (our unpublished data). Stochastic effects, chromatin folding, transcript transport/motility, and differences in timing of mitosis may have caused the fluctuations. H2A-GFP reporter studies indicated that the mitotic index of the leading hyphae is different. This was implied from the observation that the relative number of elongated GFP stained structures (representing nuclei that undergo mitotis) was different between the hyphae (Figure 1c). Differences in RNA composition may not only be related to cell cycle-dependent expression, it may also be due to a sharp exit of RNA from the nuclei during mitosis. This is suggested from the fact that nuclear pore complexes partially disassemble during mitosis in *A. nidulans *[18], thus abolishing the permeability barrier of the nuclear membrane as is found in other eukaryotes [19].

By hybridizing RNA from 1,000 hyphal tips with a *18S *rRNA probe, it was shown that the first 100 μm of the tip region of exploring hyphae of *A. niger *contains 1 pg of RNA. It is known that a typical mammalian cell contains about 10 to 30 pg total RNA [20], whereas the smaller *Escherichia coli *cells contain about 5.6 fg RNA [21]. The amount of RNA in fungal hyphae or yeast cells was not yet established. *Saccharomyces cerevisiae *has been reported to contain 60,000 mRNAs per cell [22]. Assuming that these mRNAs comprise 5% of the total RNA [20,23] and that the average RNA length is 2,500 nucleotides [20], *S. cerevisiae *would contain 2.5 pg total RNA per cell. This amount is well in line with the 1 pg of RNA that was extracted from the hyphal tip region of *A. niger*. The low amount of RNA within a cell requires amplification to microgram quantities to enable hybridization of DNA microarrays. We used the Ribo-SPIA Technology developed by Nugen. This technology gives the most reliable results when compared to other amplification protocols [24] (our own unpublished results). As a consequence of the Ribo-SPIA Technology, rRNA is also amplified.

RNA profiles were determined for the tip region of five neighboring hyphae at the most outer part of a colony of *A. niger*. This was done in two amplification experiments. The cDNA amplicons of one of the experiments had a length of 100 to 300 bp, whereas those of the other experiment were 50 to 100 bp in length. Hybridization of Affymetrix GeneChip *A. niger *Genome Arrays revealed that the higher amplicon length was accompanied by a lower number of genes with a present call (5.8 to 6.7% versus 4.1 to 4.3% for the small and large amplicons, respectively). The number of genes with a present call is low (4.1 to 6.7%) when one considers that about 50% of the genes are expressed in a sandwiched colony of *A. niger *[7]. Genes that were lowly expressed at the periphery of the colony [7] or within a pool of hyphal tips from exploring hyphae [14] often had an absent call in the arrays of the single hyphae. Apparently, RNA of lowly expressed genes is not sufficiently amplified when one uses the RNA of a single hypha. As a consequence of the low number of probe sets with a present call, higher scale factors are obtained when compared to hybridization experiments with RNA from the whole colony. Furthermore, scale factors are influenced by minimal differences in the number of present calls between the samples. Taking these facts into account, the difference in scale factors between the arrays of the single hyphae (more than three-fold but less than five-fold) was considered to be low enough to normalize and analyze the samples as a whole using the RMA method to normalize between slides. Statistical analysis indicated that this was justified (see Materials and methods).

In total, 2,608 probe sets had a present call in at least one of the five individual hyphae. These probe sets were found to comprise tRNAs, rRNAs and all 19 class I FunCats. For each functional gene category, at least 10 to 30% of the probe sets had a present call, indicating that all categories were evenly well detected. Heterogeneity between the five individual exploring hyphae was assessed by testing for overrepresentation of functional gene categories within the pool of genes with a present call within each of the single hyphae. The test revealed that genes encoding rRNAs and tRNAs were overrepresented in all five hyphae, while genes involved in ribosome biogenesis were overrepresented in four out of the five hyphae. The other nine overrepresented categories were found in one or two of the five hyphae. Heterogeneity in RNA composition was also indicated by the finding that 308 out of the 2,608 probe sets had a relatively high standard deviation (> 0.5) of the log2 RMA signal values. Apparently, expression of at least 12% of the genes is heterogeneous between neighboring exploring hyphae. This set of genes comprises all functional gene categories, except for the ones that have relatively few members. Hierarchical clustering of the 308 probe sets showed that each single hypha had a cluster of genes with higher signals when compared to the other four hyphae. Ribosome biogenesis and tRNAs were overrepresented in the majority of the samples. One hypha showed no overrepresented categories, whereas another hypha showed overrepresentation for 7 of the 10 found categories. Two of these enriched categories were involved in energy, implying that this hypha might have been metabolically more active than the other hyphae. Heterogeneity within the five individual hyphae was also assessed by selecting the top 100 genes with the highest hybridization signal in each of the hyphae. A total of 207 different genes was found in this selection, of which 43 were found in all 5 hyphae and 119 were found exclusively in one of the hyphae. For instance, all 5 hyphae contained the 4 rRNAs (*5S, 5.8S, 18S *and *28S*), 4 tRNAs and 14 genes involved in metabolism in their top 100. In contrast, 13 tRNAs, 7 genes encoding cytoplasmic ribosomal proteins and *glaA *and *eglB *were present in the top 100 of only one of the hyphae. The gene *glaA *and *18S *rRNA were also found in the list of 308 genes that showed a standard deviation > 0.5 between the log2 RMA signal values. Heterogeneity in the RNA levels of these genes as well as the *actin *gene was confirmed by QPCR. It was shown that the standard deviation of Ct values for these genes in cDNA of the five single hyphae was larger than those found in biological replicates of cDNA from pools of 100 tips or from cDNA from the whole periphery. The different yields of RNA obtained from the three sample types (that is, single hyphae, pools of 100 hyphae, and mycelium of the periphery) may have contributed to the variation in standard deviation of the Ct values. However, the conclusion that the RNA composition between individual hyphae is heterogeneous still holds and is supported by the microarray data of this study as well as our previous findings with GFP reporter studies and *in situ *hybridizations [10,11,17].

The apical region from which the transcriptome was analyzed represented half of the first hyphal compartment of the exploring hyphae of the colony. Acridine orange staining [14] and *in situ *hybridizations using *18S *rRNA as a probe [11,17] indicate that the selected apical region represents the part of the colony that is most rich in RNA. A high concentration of RNA has been associated with a high growth rate in *S. cerevisiae *[25]. Levels of rRNA, ribosomal proteins and ribosomes rapidly change when the growth rate of *S. cerevisiae *and *Neurospora crassa *changes [25-29]. This study showed that the composition of the RNA pool at the hyphal tip is heterogeneous. This heterogeneity does not seem to affect hyphal extension but does have an effect on protein secretion and possibly other cellular activities as well [10,11].

## Conclusions

We performed the first single cell transcriptome analysis of a microbe. It is shown that hyphae that experience identical environmental conditions are heterogeneous with respect to RNA composition. It is thus demonstrated that there is a disconnection between the average gene expression of the hyphae in a particular zone of a colony and the gene expression of a single hypha within such a zone. Therefore, single cell transcriptome analysis with high spatiotemporal resolution should be performed to give an accurate understanding of the processes within a cell. The RNA extraction and amplification protocol that we have developed can be used to provide such expression profiles of single hyphae (or parts thereof) that grow saprobically or that have established a parasitic or mutually beneficial symbiosis with another organism.

## Materials and methods

### Strain and growth conditions

Strain AR9#2 of *A. niger *was used in this study. This strain is a derivative of strain AB4.1 (*pyrG, cspA1*) [30] in which the construct pAN52-10S65TGGPn/s was introduced [31]. This construct contains *sGFP*(S65T) under the regulation of the *glaA *promoter of *A. niger*. To visualize nuclei, CB-A119.1 was used. This strain is a derivative of N593 (*pyrA*, cspA) [32] in which construct pCB034 was introduced according to previously described protocols [33]. Construct pCB034 contains *sGFP*(S65T) fused to *H2B *under the regulation of the constitutive *gpdA *promoter. Strains were cultured as a sandwiched colony at 30°C in the light. To this end, the fungus was grown between a perforated polycarbonate (PC) membrane (diameter 76 mm, pore size 0.1 μm; Osmonics, GE Water Technologies, Trevose, PA, USA) and a Lumox membrane (diameter 76 mm; Greiner Bio-One, Frickenhausen, Germany) [11]. The PC membrane was placed on top of solidified (1.5% agar) minimal medium [34] containing 25 mM maltose as a carbon source. Freshly harvested spores (1.5 μl of a solution of 0.8% NaCl and 0.005% Tween-80 containing 10^8 ^spores ml^-1^) were placed in the center of the PC membrane. The droplet was allowed to dry, after which the Lumox membrane was placed on top of the PC membrane with its hydrophobic side facing the inoculum.

### Calcufluor white staining and GFP fluorescence

After removing the Lumox membrane from the sandwiched colony, part of the periphery of the mycelium (with its underlying membrane) was cut with a scalpel and transferred to a glass slide. In the case of calcafluor white (CFW) staining, the sample was fixed with 70% ethanol and dried at room temperature. CFW staining was done using PBS containing 0.01% Fluorescent Brightener 28 (Sigma F-3543, St Louis, MO, USA). After staining for 1 minute, the sample was washed once with PBS. For monitoring of GFP fluorescence, samples were submerged in 90% glycerol in PBS. A Zeiss Axioscope 2PLUS (Carl Zeiss, Germany) equipped with a HBO 100 W mercury lamp, a Leica LFC 420 C camera (2,592 × 1,944 pixels) and standard DAPI and FITC filters was used to monitor fluorescence of CFW and GFP, respectively. Images were handled with Leica Application Suite software (v.2.8.1).

### Laser micro-dissection and pressure catapulting

After removing the Lumox membrane from the sandwiched colony, the mycelium and the underlying PC membrane were cut with a scalpel and part of the periphery of the colony was placed upside down onto a nucleotide and RNAse free glass slide. The PC membrane, now facing the air, was removed and the mycelium was fixed with 70% ethanol and air dried. The hyphal tips were isolated by laser pressure catapulting (LPC) using the PALM CombiSystem (Carl Zeiss MicroImaging, Germany) (Additional file 1). This system was equipped with an Axiovert 200 M Zeiss inverted microscope (Carl Zeiss, Germany) and a 3CCD color camera (HV-D30, Hitachi Kokusai Electric, Japan). The PALM CombiSystem was operated with PALM RoboSoftware v.4.0 (Carl Zeiss MicroImaging, Germany). The autoLPC option was routinely used in combination with a 40× objective. Hyphal material was catapulted into lids of 0.5 ml Eppendorf tubes that contained 50 μl RNA*later *(Qiagen, Hilden, Germany).

### RNA isolation and amplification

Three types of RNA samples were isolated from 7-day-old sandwiched colonies of *A. niger*. First, five samples were obtained each containing the RNA from a single hyphal tip; to this end, neighboring hyphae were selected. Second, three samples were obtained each containing the RNA of a pool of 100 neighboring hyphal tips; different colonies were used for this biological triplicate. Third, three samples were obtained from the outer 3-mm region of the sandwiched colony. Each sample was obtained from a different colony. RNA was isolated from these 11 samples. To this end, hyphal material that was collected in 50 μl RNA*later *was transferred to a 2-ml Eppendorf tube by a quick centrifugation step (Additional file 1). After snap-freezing in liquid nitrogen, two pre-cooled metal bullets (4.76 mm in diameter) were added and samples were ground in a Micro-Dismembrator U (B Braun Biotech, Melsungen, Germany) in a chilled container at 1,500 rpm for 60 s. The frozen material was taken up in 250 μl Trizol Reagent (Invitrogen, Carlsbad, CA, USA) by vortexing. After removing the metal bullets, 200 μl chloroform was added. After mixing well, samples were centrifuged at 10,000 g for 10 minutes. The water phase (approximately 200 μl) was mixed with 700 μl RLT from the RNeasy MinElute Cleanup Kit (Qiagen) to which 143 mM β-mercaptoethanol was added. RNA was purified following the instructions of the manufacturer and eluted with 12 μl RNAse free water. RNA samples were amplified using the WT-Ovation One-Direct RNA Amplification System (Nugen). The quality and quantity of the cDNA samples were checked using a Bioanalyzer (Agilent Technologies) and a Nanodrop (Nanodrop Technologies, Wilmington, DE, USA), respectively.

### Quantification of RNA in exploring hyphae

RNA from 1,000 tips of exploring hyphae (3 to 4 μm in width and 100 μm in length) was spotted onto a Roti-Nylon plus membrane (Roth, Karlsruhe, Germany) together with a series of RNA with known concentration. After cross-linking with UV light, the RNA was hybridized overnight at 42°C [35] with α-^32^P-CTP labeled random primed probe of *18S *rDNA. The blot was exposed to X-OMAT Blue XB films (Kodak, NY, USA) in a BioMax cassette with a BioMax TranScreen-HE (Kodak) at -80°C.

### QPCR analysis on amplified samples

QPCR was performed using the ABI Prism 7900HT SDS and SYBR Green chemistry (Applied Biosystems, Carlsbad, CA, USA). Ct levels were measured for *18S *rRNA and for mRNA of the *glaA *and *actin *gene. Primers were designed according to the recommendations of the PCR master-mix manufacturer (Applied Biosystems). Ct levels of *actin *were determined with the primers QPCRactFW1 (GTTGCTGCTCTCGTCATT) and QPCRactRV1 (AACCGGCCTTGCACATA) and those of *18S *rRNA were determined with primers QPCR18SFW1 (GGCTCCTTGGTGAATCATAAT) and QPCR18SRV1 (CTCCGGAATCGAACCCTAAT). These products had an amplification efficiency of 2. cDNA of *glaA *was amplified using primers QPCRglaAFW3 (GCACCAGTACGTCATCAA) and QPCRglaARV3 (GTAGCTGTCAGATCGAAAGT) with an amplification efficiency of 1.98. QPCR reactions were performed using 1 ng cDNA of samples amplified from RNA extracted from single hyphae (1 pg RNA), 100 hyphae (100 pg RNA) and peripheral mycelium from which the RNA was diluted towards 5 pg RNA prior to amplification.

### Microarray analysis

Amplified cDNA (5 μg; see above) was fragmented via combined chemical and enzymatic fragmentation using the protocol of the Encore Biotin Module (Nugen). The fragments were biotin-labeled to the 3-hydroxyl end using the same module following the instructions of the manufacturer. The labeled cDNA was hybridized to Affymetrix GeneChip *A. niger *Genome Arrays. The GeneChip Hybridization, Wash and Stain Kit (Affymetrix) was used for the hybridizations according to the protocol of the manufacturer with the modification that the hybridization cocktail was prepared according to the Encore Biotin Module and that the hybridization time was extended to 40 hours as recommended by Nugen. The MAS5.0 algorithm (Affymetrix) was used for quality control of the hybridized arrays. Summarized expression values of the single hypha samples were calculated using log-scale RMA [36]. The array data have been deposited in NCBI's Gene Expression Omnibus [37] and are accessible through series accession number [GEO:GSE25497] [38].

RNA was extracted in two batches (that is, hyphae 1 to 3 and hyphae 4 and 5). The batch effect on the expression values of individual genes was inferred using a standard model II ANOVA [39]. After correcting the *P*-values for false discoveries [40], it was found that all perfect match probes showed a significant batch effect with q-values ≤ 0.05 before normalization. After background subtraction, normalization and summarization, 2% of the probe sets showed a batch effect with *P*-values ≤ 0.01. After false discovery rate correction only three probe sets showed a batch effect with q-values ≤ 0.05. Taken together, it is concluded that the batch effect was negligible in the normalized data.

PCA and hierarchical clustering of probe sets and samples was performed on the Z-scores (equal to (Value-Average)/Standard deviation) derived from the log2 RMA data. Hierarchical clustering was performed in Spotfire Decision Site 7.3 software [41] using complete linkage as clustering method and 1-Pearson correlation as distance measure (d). Different subsets were tested for overrepresentation of FunCats [42] and non-FunCat categories with all 14,455 probe sets as background using a hyper-geometrical test [43]. The classification of the *A. niger *genes in FunCat categories has been described [44].

## Abbreviations

bp: base pair; CFW: calcofluor white; Ct: cycle threshold; GFP: green fluorescent protein; LPC: laser pressure catapulting; PBS: phosphate-buffered saline; PC: polycarbonate; PCA: principal component analysis; QPCR: quantitative polymerase chain reaction; RMA: robust multi-array analysis.

## Competing interests

The authors declare that they have no competing interests.

## Authors' contributions

CdB designed and performed the experiments. CdB, OB, MJJ, TMB, and HABW analyzed the results. All authors were involved in drafting the manuscript and read and approved its final version.

## Supplementary Material

Additional file 1 **A protocol describing the procedure to extract and amplify RNA of single hyphae (or parts thereof)**. Click here for file

Additional file 2 **A figure showing the amount of RNA within 1,000 hyphae**. Click here for file

Additional file 3 **A table, similar to Table **1**listing Ct values of a QPCR analysis of the two amplification experiments of RNA from single hyphae (hyphae 1 to 3 and 4 and 5)**. Click here for file

Additional file 4 **A table listing Affymetrix quality control checks after hybridizing amplified cDNA from single hyphal tips**. Click here for file

Additional file 5 **Scatter plots that show that genes with an absent call in the single hypha analysis are generally lowly expressed in a transcriptome analysis of a population of hyphae from the same zone of the colony**. Click here for file

Additional file 6 **A table listing the probe sets that gave a present call in at least one of the five arrays of a single hypha**. Click here for file

Additional file 7 **A table listing the overrepresentation of functional gene categories within the probe sets with a present call in each of the individual hyphae**. Click here for file

Additional file 8 **A table listing the probe sets with a present call in at least one of the five hyphae and a standard deviation > 0.5 between the log2 RMA signals of the single hyphae**. Click here for file

Additional file 9 **A figure showing *P*-values of the functional gene categories in Table **4**after analyzing hyphae 1 to 5 and hyphae 1 to 3 and 4 and 5 separately**. Click here for file

Additional file 10 **A table listing the top 100 genes with the highest signal values in each of the five hyphae**. Click here for file

Additional file 11 **A table listing the genes with the highest signal values that can be found in the top 100 of 2, 3, 4 or 5 out of the 5 single hyphae**. Click here for file

Additional file 12 **A table listing the genes with the highest signal values that can be found in the top 100 of only 1 out of the 5 single hyphae**. Click here for file
