# Supplementary material for: Single cell transcriptomics of neighboring hyphae of Aspergillus niger
Source: Genome Biol. 2011 Aug 4;12(8):R71. doi: 10.1186/gb-2011-12-8-r71 (PMC3245611; doi:10.1186/gb-2011-12-8-r71)
Supplement: Additional file 1 — A protocol describing the procedure to extract and amplify RNA of single hyphae (or parts thereof). [file gb-2011-12-8-r71-S1.DOC]

**Additional data file 1**

**Protocol for (sub)cellular whole transcriptome analysis in *Aspergillus niger***

The protocol includes:

1. Colony growth and sample preparation
2. Harvesting hyphae using LPC technology
3. RNA isolation
4. RNA amplification
5. cDNA labeling

**1.** **Colony growth and sample preparation**

Hyphal material is harvested using LPC (see below). For this, colonies need to be grown in a (near) 2 dimensional way. To establish this, colonies are normally grown in a thin agarose layer in between perforated PC membranes (Wöst*en et a*l., 1991). However, the agarose layer precludes dissection of the hyphae. Therefore, mycelium was grown directly in between membranes:

- Petri dishes (9 cm in diameter) are filled with 20 ml medium containing 1.5% agarose.
  - The plate is dried (without lid) at 60 °C for 10 min after the medium has solidified.
- A sterile polycarbonate (PC) membrane (Profiltra, Netherlands) with 0.1 μm pores and a diameter of 76 mm is placed on top of the medium.
  - PC membranes are sterilized between wet paper in a glass Petri dish. They are dried at 60 °C before use with the lid kept on the container.
- 1.5 μl of a fresh *A. niger* spore suspension (10^8^ spores ml^-1^ 0.8% NaCl, 0.005% Tween-80) or a small piece of mycelium is used as an inoculum. In the case a spore solution is used, excess of water is removed by drying for 8 to 16 hours at room temperature (with lid on Petri dish) before the Lumox foil (see below) is placed on top of the inoculum.
- Sterile Lumox foil (Greiner Bio-One, Germany) with a diameter of 76 mm (cut from a piece of 5 m x 305 mm) is placed on the PC membrane with the hydrophobic side of the foil facing the inoculum.
- Colonies are grown for 7 days at 30 ^0^C in a water saturated growth chamber.
- The Lumox foil is removed from the colony with a pair of forceps. The colony (and the underlying PC membrane) is cut in 12 parts (“pie pieces”) with a scalpel.
  - Because the hyphae harvested from these slides will be used for RNA isolation and amplification it is important that small equipment is cleaned with RNaseZAP (Ambion, TX) and DNA-OFF (MP Biomedicals, OH) prior to sample preparation.
  - Note that the colony will stick to the PC membrane and not to the Lumox foil.
- Clean a microscope object glass with RNaseZAP and DNA-OFF. Transfer colony pieces to these slides using the PC membrane underlying the mycelium as a carrier.
- Place sample gently upside down on the slide (*i.e.* mycelium first; the PC membrane is now positioned on top of the colony part).
- Peel the PC membrane off starting from the central part of the colony towards the periphery. In this way the peripheral hyphae will maintain their position.
- Fix the sample on the slide by wetting the mycelium with 70% ethanol (about 200-500 μl). Air-dry the sample. This fixation procedure results in the isolation of intact total RNA.

Note: PEN-membrane slides (Carl Zeiss Micro Imaging, Germany) are generally used for laser dissection. These slides, function as a sample carrier after micro-dissection. In this protocol glass slides are used. Using glass slides, hyphal tips cannot be dissected and catapulted as a whole, but have to be catapulted in pieces. Fragmentation of the hyphal material leads to improved homogenization in combination with the dismembrator (See section 3. RNA isolation) and, as a consequence, in more reproducible RNA isolation.

**2. Harvesting hyphae using LPC technology**

Hyphae are harvested with a PALM CombiSystem (Carl Zeiss Micro Imaging) equipped with an Axiovert 200M Zeiss inverted microscope (Carl Zeiss AG, Germany) and a 3CCD color camera (HV-D30, Hitachi Kokusai Electric, Japan). Prior to laser pressure catapulting (LPC) the slide holder and the capture devise are cleaned with RNaseZAP and DNA-OFF (these parts of the instrument will be in direct contact with the samples).

- Pipet 50 µl RNAlater (Qiagen, Germany) in the cap of a sterile 0.5 ml Eppendorf tube. This avoids RNA degradation within the catapulted hyphal material.
- Place cap in the capture device. Position cap above the slide containing the mycelial sample.
- Hyphae are catapulted into the Eppendorf cap using a 40x magnification and the auto LPC option. In order to harvest, draw an area around the hypha of choice with the draw tool. The auto LPC option will fill this area with dots. These dots mark the spots where the laser will give pulses. In this way hyphal fragments are catapulted into the Eppendorf cap.
  - Make sure the sample is in focus (adjustment of the objective) and the right percentage of the laser power is used. Adjust the laser settings using a piece of the sample you are not planning to use for harvesting. The laser power must be adjusted such that all of the mycelium is catapulted up while only a discrete dot is punctured in the glass slide underneath the sample.
- The cap containing RNAlater and the harvested hyphal material is taped on a 2 ml conical Eppendorf tube in which homogenization will take place. The RNAlater and the hyphae are transferred to the 2 ml tube by centrifugation for 5 sec in an Eppendorf centrifuge (10000 rpm).
  - Note that transferring is not done by pipetting. This is to minimize the risk that hyphal material is lost by sticking to the pipet tip.
- The cap is removed and the sample is snap frozen in the 2 ml Eppendorf tube using liquid nitrogen.

**3. RNA isolation**

Make sure that work space and equipment is RNAse and DNA free by cleaning with RNaseZAP and DNA-OFF. Use clean pipettes that are not used for GMO work in combination with nuclease free filter tips (Axygen, CA). The Qiagen RNA MinElute Cleanup kit is used for purification.

- Two new metal balls (that had been cooled with liquid nitrogen), 4.76 mm in diameter, are added to the 2 ml Eppendorf tube with frozen sample.
- Samples are ground in a Micro-Dismembrator U (B. Braun Biotech, Germany) in a chilled container for 3 disposable test tubes of 2,2 ml (cat. no. 8531889, B.Braun Biotech) at 1500 rpm for 60 sec.
- Add 250 µl TRIzol (Invitrogen, CA) and vortex until sample is thawed and suspended. Remove the metal balls using a magnet and leave sample at room temperature for 5 min. Note that the metal balls are removed after the homogenized sample is dissolved in Trizol to make sure that hyphal material is not lost. Do not re-use the metal balls.
- Add 200 µl chloroform, vortex and leave sample at room temperature for 3 min.
- Centrifuge for 10 min at 10.000 g in an Eppendorf centrifuge and transfer water phase (upper phase; usually around 150-200 µl) to a new 2 ml Eppendorf tube. The water phase is well mixed with 525-700 µl RLT from the RNeasy MinElute Cleanup Kit (Qiagen) to which 143 mM β–mercaptoethanol is added (according to the protocol provided by the manufacturer).
- Add 500 µl 96-100% ethanol and mix well by pipetting. Immediately continue with the next step.
- Transfer aliquots of up to 700 µl to an RNeasy MinElute spin column placed in a 2 ml collection tube. Close the lid gently and centrifuge for 15 sec at 10.000 rpm. Discard the flow-through.
- Place the spin column in a new collection tube and wash the column membrane by adding 500 µl RPE buffer. Close the lid gently and centrifuge for 15 sec at 10.000 rpm. Discard the flow-through.
- Add 500 µl freshly made 80% ethanol (v/v) to the spin column, close the lid gently, and centrifuge for 2 min at 10.000 rpm to wash and dry the column membrane.
- Place the spin column in a new 2 ml collection tube, open the lid and centrifuge at full speed for 5 min. This eliminates possible carry-over of ethanol and residual flow-through remains on the outside of the spin column.
- Place the spin column in a new 1.5 ml collection tube. Add 12 µl RNase-free water directly to the membrane (dead volume of the column is 2 µl). Close the lid gently and leave at room temperature for 5 min. Elute the RNA sample from the column membrane by centrifugation for 1 min at 10.000 rpm.

**4. RNA amplification**

Isolated RNA is amplified using the WT-Ovation One-Direct RNA Amplification System (Nugen, CA). According to the supplier, 10-500 pg input RNA results in microgram quantities of cDNA amplicons with a length of about 50 to 500 bases. The RNA sample is eluted in 10 µl. Note that only a 5 µl volume can be used for the amplification. Precipitation using a carrier prior to amplification is not recommended because the carrier might interfere with downstream reactions. Therefore, realize that only half of the eluted RNA sample can be used for amplification.

- Prior to RNA amplification, make sure workspace and equipment is RNAse and DNA free by cleaning with RNaseZAP and DNA-OFF. Use clean pipettes that are not used for GMO work in combination with filter tips (Axygen).
- Amplification protocols provided by Nugen are followed up to every detail.
- Amplified cDNA is purified using the QIAQuick PCR Purification kit (Qiagen) en eluted in 30 µl H2O provided in the Nugen kit.
- Concentration is measured using 2 μl sample and a Nanodrop (Nanodrop Technologies, DE) with the ssDNA option.
- Amplification is checked using a Bioanalyser in combination with the RNA 6000 Nano Assay (Agilent Technologies, CA) following the protocol provided by the manufacturer. Normally 25 to 500 ng of RNA is used in this assay. If micrograms of cDNA are obtained after amplification, 1 µl of sample is sufficient for the quality check with the Bioanalyzer.

**5. cDNA labeling**

5 μg of amplified cDNA is fragmented and labeled using the Encore Biotin Module (Nugen). This results in cDNA suitable for hybridization of Affymetrix GeneChip arrays.
