# Supplementary material for: Single cell transcriptomics of neighboring hyphae of Aspergillus niger
Source: Genome Biol. 2011 Aug 4;12(8):R71. doi: 10.1186/gb-2011-12-8-r71 (PMC3245611; doi:10.1186/gb-2011-12-8-r71)
Supplement: Additional file 2 — A figure showing the amount of RNA within 1,000 hyphae. [file gb-2011-12-8-r71-S2.DOC]

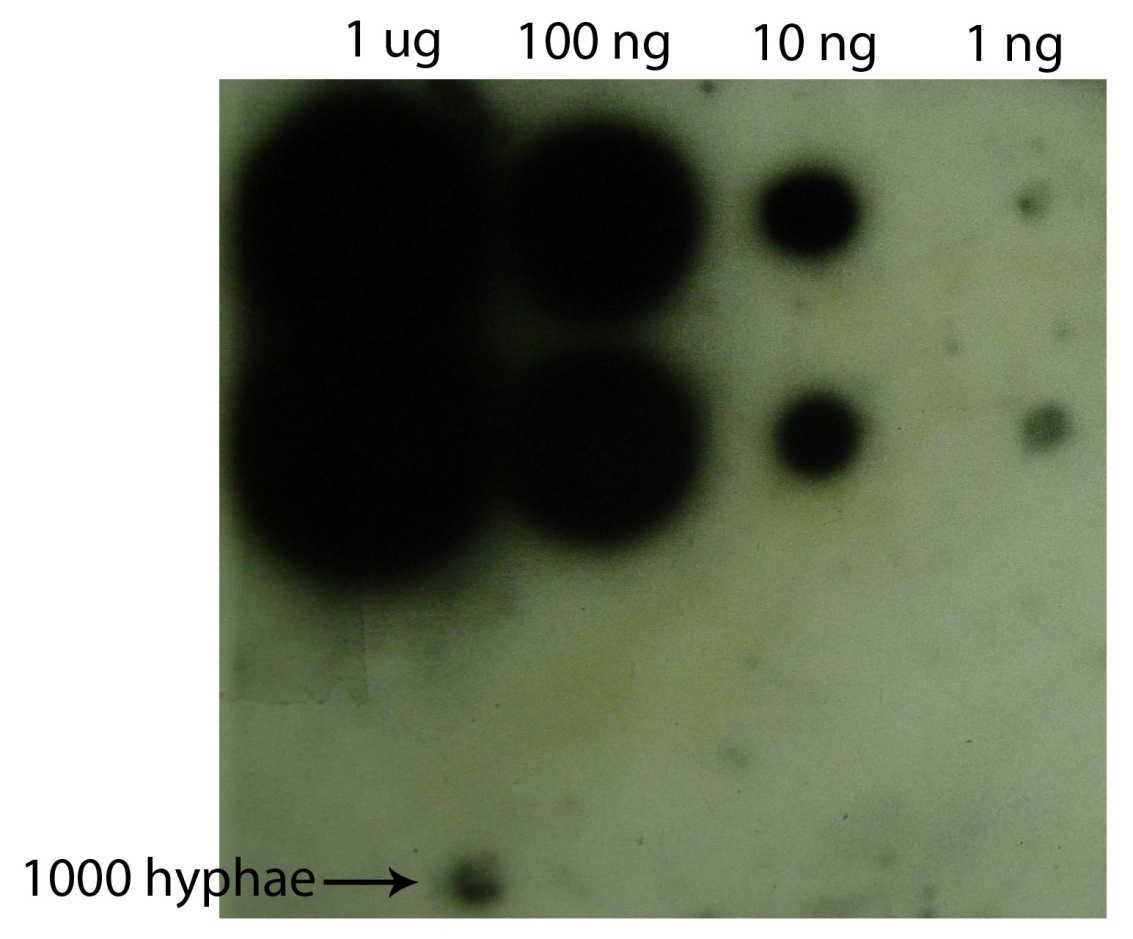


**Additional data file 2.** *18S* rRNA hybridization of a dot blot containing total RNA of 1000 hyphal tips and RNA samples with known quantity. Hybridization shows that 1000 hyphal tips contain about 1 ng total RNA.
