# Supplementary material for: Single cell transcriptomics of neighboring hyphae of Aspergillus niger
Source: Genome Biol. 2011 Aug 4;12(8):R71. doi: 10.1186/gb-2011-12-8-r71 (PMC3245611; doi:10.1186/gb-2011-12-8-r71)
Supplement: Additional file 3 — A table, similar to Table 1listing Ct values of a QPCR analysis of the two amplification experiments of RNA from single hyphae (hyphae 1 to 3 and 4 and 5). [file gb-2011-12-8-r71-S3.DOC]

**Additional data file 3**

**Transcript accumulation is heterogenic between hyphae at the periphery of an *A. niger* colony.** QPCR was performed using 1 ng of cDNA amplified from RNA of tips of single hyphae from the outer periphery of a 7-days-old sandwiched colony, from RNA of a pool of 100 of such tips, and from RNA of the 3-mm-wide periphery of sandwiched colonies of *A. niger*. For the single hyphae, samples were analyzed as a whole (hypha 1-5) and as separate amplification experiments (hypha 1-3 and hypha 4-5). The average threshold (µ Ct) and their standard deviations () are given for *18S* rDNA and the *actin* and *glaA* genes*.*  technical represents the range in standard deviations obtained for the 6 technical replicates for each of the biological replicates. * indicates that the standard deviation of the biological replicates is significantly higher than the maximum standard deviation of the technical replicates within a sample type (p≤0.01).

| **Gene** | **Sample type** | **µ Ct** | **σ** | **σ technical** |
| --- | --- | --- | --- | --- |
| *18S* | hypha 1-5 | 20.23 | 2.40 | 0.53-1.26 |
|  | hypha 1-3 | 17.96 | 1.10 | 0.75-1.03 |
|  | hypha 4-5 | 22.10 | 1.48 | 1.02-1.26 |
|  | 100 hyphae | 17.39 | 0.42 | 0.26-0.43 |
|  | 5 pg periphery | 12.58 | 0.28 | 0.15-0.35 |
| *actin* | hypha 1-5 | 30.30 | 6.07* | 0.11-1.03 |
|  | hypha 1-3 | 27.81 | 5.49* | 0.11-0.33 |
|  | hypha 4-5 | 32.35 | 7.26* | 0.11-1.03 |
|  | 100 hyphae | 28.15 | 4.80 | 0.12-1.71 |
|  | 5 pg periphery | 18.05 | 0.57 | 0.09-0.22 |
| *glaA* | hypha 1-5 | 25.47 | 4.63* | 0.06-0.66 |
|  | hypha 1-3 | 21.53 | 2.07* | 0.08-0.10 |
|  | hypha 4-5 | 27.22 | 3.21* | 0.06-0.13 |
|  | 100 hyphae | 24.51 | 2.22 | 0.10-0.22 |
|  | 5 pg periphery | 18.65 | 0.49 | 0.10-0.14 |
