# Supplementary material for: Single cell transcriptomics of neighboring hyphae of Aspergillus niger
Source: Genome Biol. 2011 Aug 4;12(8):R71. doi: 10.1186/gb-2011-12-8-r71 (PMC3245611; doi:10.1186/gb-2011-12-8-r71)
Supplement: Additional file 4 — A table listing Affymetrix quality control checks after hybridizing amplified cDNA from single hyphal tips. [file gb-2011-12-8-r71-S4.DOC]

**Additional data file 4.** Affymetrix quality control checks after hybridizing amplified cDNA from single hyphal tips. RNA from hyphae 1-3 and hyphae 4-5 were amplified on different days.

| Sample | Scale Factor | Percentage present calls | Number of probe sets with a present call |
| --- | --- | --- | --- |
| Hypha 1 | 4.7 | 5.8 | 951 |
| Hypha 2 | 3.9 | 6.7 | 1101 |
| Hypha 3 | 6.6 | 6.1 | 975 |
| Hypha 4 | 16.9 | 4.1 | 668 |
| Hypha 5 | 15.3 | 4.3 | 684 |
