# Supplementary material for: Single cell transcriptomics of neighboring hyphae of Aspergillus niger
Source: Genome Biol. 2011 Aug 4;12(8):R71. doi: 10.1186/gb-2011-12-8-r71 (PMC3245611; doi:10.1186/gb-2011-12-8-r71)
Supplement: Additional file 5 — Scatter plots that show that genes with an absent call in the single hypha analysis are generally lowly expressed in a transcriptome analysis of a population of hyphae from the same zone of the colony. [file gb-2011-12-8-r71-S5.DOC]

**
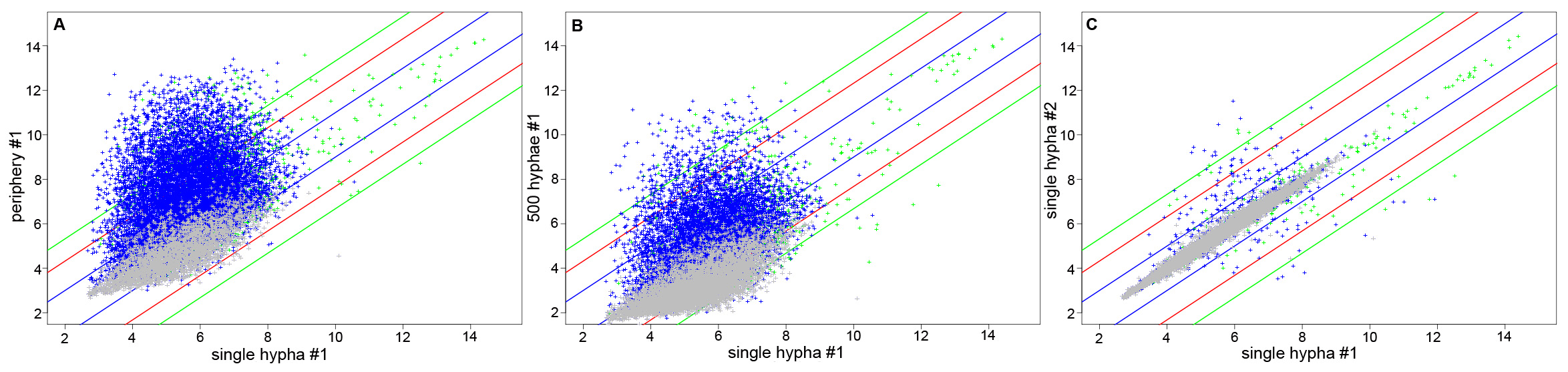
**

**Additional data file 5.** Scatter plots depicting present and absent calls of all probe sets of an array of a single hyphal tip and of an array of the periphery of the colony (A); an array of a single hyphal tip and of an array of a pool of 500 hyphal tips (B) and two arrays of different single hyphal tips (C). Probe sets with an absent call in an array of a single hyphal tip have generally low signal values in arrays of RNA from the periphery or from a pool of 500 hyphal tips. Green = present call in both samples, Blue = present call in one of the samples, and Grey = absent call in both samples.
