# Supplementary material for: Single cell transcriptomics of neighboring hyphae of Aspergillus niger
Source: Genome Biol. 2011 Aug 4;12(8):R71. doi: 10.1186/gb-2011-12-8-r71 (PMC3245611; doi:10.1186/gb-2011-12-8-r71)
Supplement: Additional file 9 — A figure showing P-values of the functional gene categories in Table 4after analyzing hyphae 1 to 5 and hyphae 1 to 3 and 4 and 5 separately. [file gb-2011-12-8-r71-S9.DOCX]

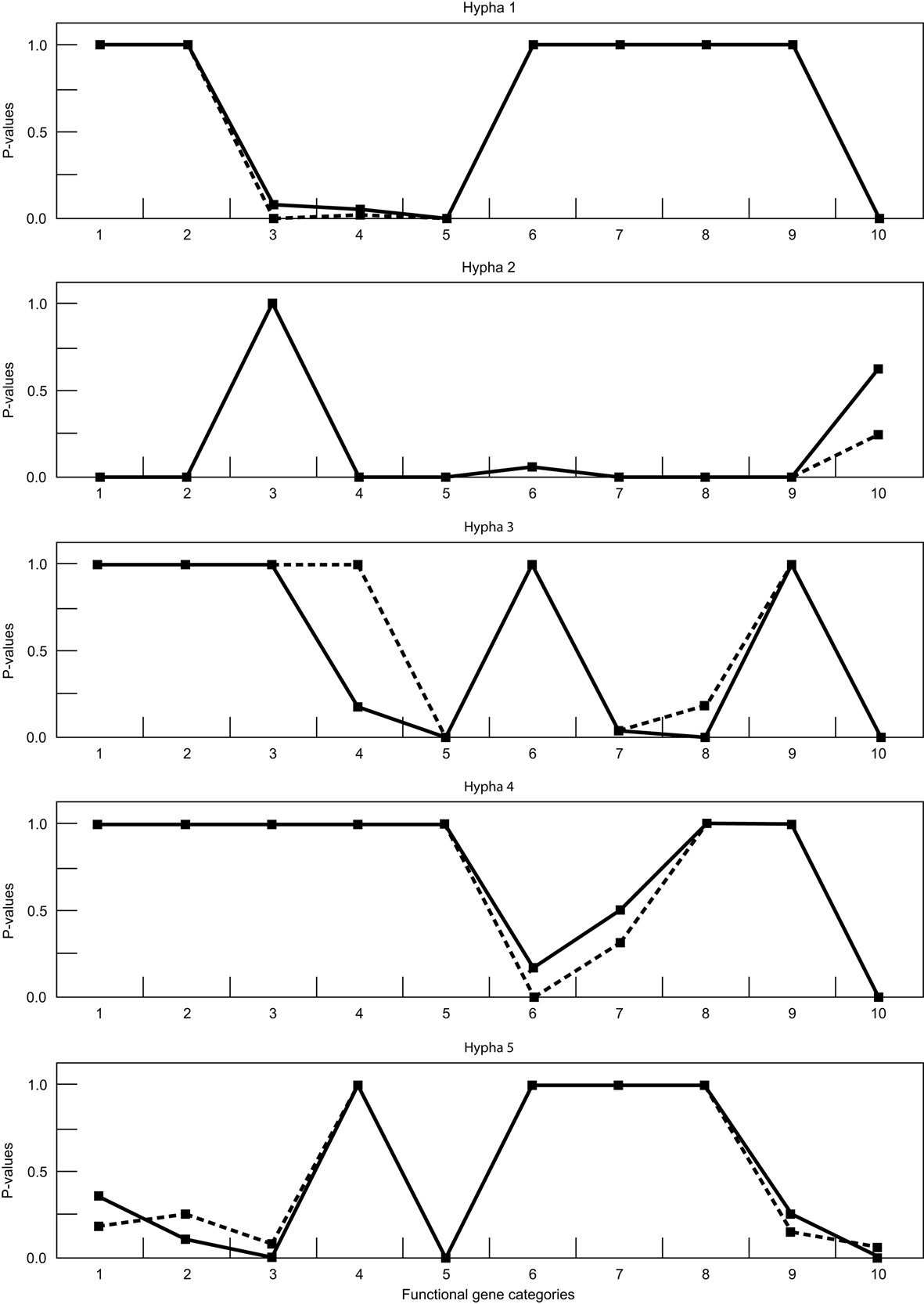


**Hypha 1**

Functional gene categories

P-values

1.000

0.500

0.000

1.000

0.500

0.000

1.000

0.500

0.000

1.000

0.500

0.000

1.000

0.500

0.000

**Hypha 2**

**Hypha 3**

**Hypha 4**

**Hypha 5**

**Additional data file 9.** Figure showing P-values of the functional gene categories in Table 4 after analyzing hypha 1-5 (dotted line) and hypha 1-3 and 4-5 separately (solid line)
