# Supplementary material for: Single cell transcriptomics of neighboring hyphae of Aspergillus niger
Source: Genome Biol. 2011 Aug 4;12(8):R71. doi: 10.1186/gb-2011-12-8-r71 (PMC3245611; doi:10.1186/gb-2011-12-8-r71)
Supplement: Additional file 11 — A table listing the genes with the highest signal values that can be found in the top 100 of 2, 3, 4 or 5 out of the 5 single hyphae. [file gb-2011-12-8-r71-S11.DOC]

**Additional data file 11 Genes with the highest signal values that can be found in the top 100 of 2, 3, 4 or 5 out of the 5 single hyphae. Unidentified proteins are listed in Additional data file 10.**

|  | **In top 100 of all hyphae** |  |
| --- | --- | --- |
| **Gene number** | **Description** | **Functional gene category** |
| An01g03480 | strong similarity to sorbitol dehydrogenase gutB - *Bacillus subtilis* | 01 metabolism |
| An04g02220 | strong similarity to L-serine dehydratase CHA1 - *Saccharomyces cerevisiae* | 01 metabolism |
| An04g02410 | strong similarity to cobalamin and cobamide biosynthesis protein COB W patent WO9111518-A - *Pseudomonas denitrificans* | 01 metabolism |
| An09g05770 | strong similarity to cytosolic exopolyphosphatase PPX1 - *Saccharomyces cerevisiae* | 01 metabolism |
| An10g00520 | weak similarity to 1-aminocyclopropane-1-carboxylate deaminase - *Pseudomonas* sp. [truncated orf] | 01 metabolism |
| An11g01390 | similarity to phosphoglycerate dehydrogenase serA - *Bacillus subtilis* | 01 metabolism |
| An11g05690 | similarity to hypothetical alpha subunit of dinitrogenase reductase nifH - unidentified nitrogen-fixing bacteria | 01 metabolism |
| An12g00670 | strong similarity to cinnamyl-alcohol dehydrogenase CAD14 – *Nicotiana tabacum* | 01 metabolism |
| An12g09340 | strong similarity to ferulic acid decarboxylase FDC1 patent EP857789-A2 - *Saccharomyces cerevisiae* | 01 metabolism |
| An15g04610 | strong similarity to xylitol dehydrogenase xdh - *Galactocandida mastotermitis* [truncated ORF] | 01 metabolism |
| An16g07090 | similarity to transmembrane protein sequence of patent WO9927105-A2 - *Chlamydia pneumoniae* | 01 metabolism |
| An17g02040 | strong similarity to the protein required for normal CLN1 and CLN2 G1 cyclin expression Ctr9 - *Saccharomyces cerevisiae* | 03 cell cycle and DNA processing |
| An02g06750 | strong similarity to DEAD box protein Dbp45A - *Drosophila melanogaster* | 04 transcription |
| An07g07270 | similarity to tRNA 2 -phosphotransferase TPT1 - *Saccharomyces cerevisiae* | 04 transcription |
| An07g09050 | similarity to hypothetical transcription regulator SC5F2A.29 - *Streptomyces coelicolor* | 04 transcription |
| An11e07520 | repetetive DNA | Repeat |
| An13e00260 | repetetive DNA | Repeat |
| An03e03150 | 5.8S ribosomal RNA | rRNA |
| An03e03180 | 28S ribosomal RNA | rRNA |
| An03e03260 | 18S ribosomal RNA | rRNA |
| An01e11490 | trnaSaga | Trna |
| An01e12510 | trnaScga | Trna |
| An03e02440 | trnaLtag | Trna |
| An08e06020 | trnaSaga | Trna |
|  | **In top 100 of 4 hyphae** |  |
| **Gene number** | **Description** | **Functional gene category** |
| An12g00950 | rhamnogalacturonase rhgA - *Aspergillus niger* | 01 metabolism |
| An14g03240 | strong similarity to acyl-CoA dehydrogenase (NADP+) ACDH - *Mycobacterium tuberculosis* | 01 metabolism |
| An18g01520 | strong similarity to quinic-acid utilisation gene qutH - *Aspergillus nidulans* | 01 metabolism |
| An17g00730 | strong similarity to electron transfer flavoprotein (ETF) beta chain - *Paracoccus denitrificans* | 02 energy |
| An01g10550 | strong similarity to transposase Minos-2 - *Drosophila hydei* | 29 transposable elements, viral and plasmid proteins |
| An01e01100 | 5S ribosomal RNA | rRNA |
| An12e07440 | trnaStga | tRNA |
|  | **In top 100 of 3 hyphae** |  |
| **Gene number** | **Description** | **Functional gene category** |
| An01g14740 | glucose oxidase precursor goxC - *Aspergillus niger* [putative sequencing error] | 01 metabolism |
| An12g08630 | strong similarity to cytochrome P450 monooxygenase TRI11 – *Fusarium sporotrichioides* | 01 metabolism |
| An02g06750 | strong similarity to DEAD box protein Dbp45A - *Drosophila melanogaster* | 04 transcription |
| An04g08980 | strong similarity to cytoplasmic ribosomal protein of the large subunit L43A - *Saccharomyces cerevisiae* | 05 protein synthesis |
| An02g04240 | weak similarity to acinusS mRNA - *Homo sapiens* | 10 cellular communication or signal transduction mechanism |
| An14g04060 | strong similarity to chloride channel 3 clcn3 - *Mus musculus* | 13 regulation of or interaction with cellular environment |
| An01g08120 | strong similarity to ACOB protein - *Aspergillus nidulans* | 25 development (Systemic) |
|  | **In top 100 of 2 hyphae** |  |
| **Gene number** | **Description** | **Functional gene category** |
| An12g03430 | similarity to glucose oxidase goxC - *Aspergillus niger* [truncated ORF] | 01 metabolism |
| An18g03470 | similarity to the tRNA-specific adenosine deaminase subunit Tad1 - *Saccharomyces cerevisiae* | 04 transcription |
| An02g13850 | strong similarity to cytoplasmic ribosomal protein of the large subunit URP1 - *Saccharomyces cerevisiae* | 05 protein synthesis |
| An08g01800 | strong similarity to hypothetical mitochondrial carrier protein AgPET8 - *Ashbya gossypii* | 08 cellular transport and transport mechanisms |
| An17g00650 | similarity to the cercosporin resistance protein Crg1 - *Cercospora nicotianae* | 11 cell rescue, defense and virulence |
