# Supplementary material for: Single cell transcriptomics of neighboring hyphae of Aspergillus niger
Source: Genome Biol. 2011 Aug 4;12(8):R71. doi: 10.1186/gb-2011-12-8-r71 (PMC3245611; doi:10.1186/gb-2011-12-8-r71)
Supplement: Additional file 12 — A table listing the genes with the highest signal values that can be found in the top 100 of only 1 out of the 5 single hyphae. [file gb-2011-12-8-r71-S12.DOC]

**Additional data file 12 Genes with the highest signal values that can be found in the top 100 of only 1 out of the 5 single hyphae. Unidentified proteins are listed in Additional data file 10.**

| **Gene number** | **Description** | **Functional gene category** |
| --- | --- | --- |
| An01g10030 | strong similarity to syringomycin-resistance gene SYR2 - *Saccharomyces cerevisiae* | 01 metabolism |
| An01g11270 | similarity to 2,3-dihydroxybenzoic acid decarboxylase patent WO9909048-A1- *Aspergillus niger* | 01 metabolism |
| An02g03730 | strong similarity to cobW protein - *Pseudomonas denitrificans* [truncated ORF] | 01 metabolism |
| An03g04280 | strong similarity to pyridoxine synthesis component pyroA - *Aspergillus nidulans* | 01 metabolism |
| An03g06550 | glucan 1,4-alpha-glucosidase glaA - *Aspergillus niger* | 01 metabolism |
| An04g03530 | strong similarity to NAD-dependent D-arabinitol dehydrogenase ARD - *Candida tropicalis* | 01 metabolism |
| An04g09550 | strong similarity to 4-Hydroxyacetophenone monooxygenase hapE - *Pseudomonas fluorescens* | 01 metabolism |
| An06g00990 | strong similarity to fumarate reductase FRDS - *Saccharomyces cerevisiae* | 01 metabolism |
| An07g01960 | strong similarity to stearoyl-CoA desaturase P-ole1 - *Pichia angusta* | 01 metabolism |
| An07g04300 | strong similarity to 3-methylcrotonyl-CoA carboxylase (MCC) biotin-containing alpha subunit MCCA - *Homo sapiens* | 01 metabolism |
| An07g08360 | similarity to pyrazinamidase or nicotinamidase pncA - *Escherichia coli* | 01 metabolism |
| An07g08710 | alpha, alpha-trehalose-phosphate synthase (UDP-forming) 2 (trehalose-6-phosphate UDP-glucose phosphate glucosyltransferase) tpsB - *Aspergillus niger* | 01 metabolism |
| An08g07380 | strong similarity to malonyl CoA synthetase MatB - *Rhizobium trifolii* | 01 metabolism |
| An09g03830 | strong similarity to 2,2-dialkylglycine decarboxylase structural protein dgdA - *Pseudomonas cepacia* | 01 metabolism |
| An09g04850 | strong similarity to p-sulfobenzyl alcohol dehydrogenase TsaC - *Comamonas testosteroni* | 01 metabolism |
| An11g04370 | strong similarity to cytochrome b5 - *Mortierella alpine* | 01 metabolism |
| An11g06230 | similarity to oxidoreductase from patent WO0100844 - *Corynebacterium glutamicum* | 01 metabolism |
| An12g04590 | similarity to dihydrofolate reductase DHFR - *Pneumocystis carinii* | 01 metabolism |
| An14g02900 | weak similarity to cellobiose dehydrogenase CDH - *Trametes versicolor* | 01 metabolism |
| An14g04050 | strong similarity to pyridoxamine-phosphate oxidase pdx3 - *Saccharomyces cerevisiae* | 01 metabolism |
| An15g03770 | strong similarity to diacylglycerol acyl transferase (MR1) of patent WO200001713-A2 - *Mortierella ramanniana* | 01 metabolism |
| An16g06800 | strong similarity to endoglucanase eglB - *Aspergillus niger* | 01 metabolism |
| An16g09070 | strong similarity to glucosamine-6-phosphate deaminase protein of patent WO9835047-A1 - *Escherichia coli* | 01 metabolism |
| An17g01150 | strong similarity to acyl-CoA dehydrogenase MCAD - *Rattus norvegicus* | 01 metabolism |
| An18g04560 | similarity to peptidoglycan GlcNAc deacetylase PgdA - *Streptococcus pneumoniae* | 01 metabolism |
| An08g06550 | strong similarity to subunit VIII of ubiquinol--cytochrome c reductase - *Saccharomyces cerevisiae* | 02 energy |
| An11g10200 | strong similarity to subunit VIa of cytochrome c oxidase COX13 - *Saccharomyces cerevisiae* | 02 energy |
| An15g00690 | strong similarity to 14.8 kD subunit of NADH:ubiquinone reductase - *Neurospora crassa* | 02 energy |
| An01g04640 | strong similarity to topoisomerase I CaTOP1 - *Candida albicans* | 03 cell cycle and DNA processing |
| An02g10450 | strong similarity to GTP-binding protein VPS1 – *Saccharomyces cerevisiae* | 03 cell cycle and DNA processing |
| An04g08710 | strong similarity to protein involved in DNA repair and recombination uvsH - *Emericella nidulans* | 03 cell cycle and DNA processing |
| An08g01090 | similarity to Ada Histone acetyltransferase complex component AHC1 - *Saccharomyces cerevisiae* | 03 cell cycle and DNA processing |
| An08g03190 | strong similarity to tubulin beta chain beta-tubulin - *Aspergillus flavus* | 03 cell cycle and DNA processing |
| An11g06990 | similarity to UV damage nucleotide excision repair protein Rph16 - *Schizosaccharomyces pombe* | 03 cell cycle and DNA processing |
| An11g11110 | strong similarity to condensin complex component cnd1 - *Schizosaccharomyces pombe* | 03 cell cycle and DNA processing |
| An14g05320 | strong similarity to cell cycle regulator p21 protein, Wos2 - *Schizosaccharomyces pombe* | 03 cell cycle and DNA processing |
| An01g07250 | strong similarity to 7.7 kD subunit of DNA-directed RNA polymerase II ABC10 alpha - *Saccharomyces cerevisiae* | 04 transcription |
| An08g06940 | strong similarity to histone H4.1 - *Emericella nidulans* | 04 transcription |
| An11g10110 | similarity to protein SRB8 - *Saccharomyces cerevisiae* | 04 transcription |
| An15g03350 | strong similarity to PalC - *Emericella nidulans* | 04 transcription |
| An16g08450 | strong similarity to hypothetical transcriptional regulator SPCC417.09c - *Schizosaccharomyces pombe* | 04 transcription |
| An02g06050 | strong similarity to cytoplasmic ribosomal protein of the small subunit S16.e - *Saccharomyces cerevisiae* | 05 protein synthesis |
| An02g13840 | strong similarity to cytoplasmic ribosomal protein of the small subunit S9 - Homo sapiens | 05 protein synthesis |
| An11g01690 | strong similarity to cytoplasmic ribosomal protein of the small subunit S30 - *Saccharomyces cerevisiae* | 05 protein synthesis |
| An11g09500 | strong similarity to cytoplasmic ribosomal protein of the small subunit S4.e -*Saccharomyces cerevisiae* | 05 protein synthesis |
| An12g04670 | strong similarity to translation initiation factor eIF-5 - Saccharomyces cerevisiae | 05 protein synthesis |
| An12g04860 | strong similarity to cytoplasmic ribosomal protein of the large subunit L30 - *Saccharomyces cerevisiae* | 05 protein synthesis |
| An13g01070 | strong similarity to 40S ribosomal protein S28.e.B RPS 28B (RPS33B) - *Saccharomyces cerevisiae* | 05 protein synthesis |
| An17g02390 | strong similarity to cytoplasmic ribosomal protein of the small subunit RP10B - *Saccharomyces cerevisiae* [putative sequencing error] | 05 protein synthesis |
| An07g02010 | strong similarity to multicatalytic endopeptidase complex chain Y7 PRE8 - *Saccharomyces cerevisiae* | 06 protein fate (folding, modification, destination) |
| An07g03750 | strong similarity to phosphatidylinositol-phosphatidylcholine transfer protein SEC14 - *Yarrowia lipolytica* | 06 protein fate (folding, modification, destination) |
| An07g03880 | serine proteinase pepC - *Aspergillus niger* [putative frameshift] | 06 protein fate (folding, modification, destination) |
| An07g08300 | cyclophilin-like peptidyl prolyl cis-trans isomerase cypH - *Aspergillus niger* | 06 protein fate (folding, modification, destination) |
| An07g09590 | strong similarity to glutathione S-transferase GST of patent US5962229-A - *Zea mays* | 06 protein fate (folding, modification, destination) |
| An15g06470 | similarity to signal sequence receptor alpha chain - *Canis lupus familiaris* | 06 protein fate (folding, modification, destination) |
| An02g06360 | similarity to arp2 or 3 complex 16kD subunit arc16 - *Homo sapiens* | 08 cellular transport and transport mechanisms |
| An12g07720 | strong similarity to ferric (and cupric) reductase FRE2 – *Saccharomyces cerevisiae* | 08 cellular transport and transport mechanisms |
| An15g01930 | similarity to integral membrane protein PTH11 - *Magnaporthe grisea* | 10 cellular communication or signal transduction mechanism |
| An14g01840 | similarity to hypothetical temperature-shock induced protein TIR3 - *Saccharomyces cerevisiae* | 11 cell rescue, defense and virulence |
| An02g08020 | strong similarity to H+-transporting ATPase lipid-binding protein vma3 - *Neurospora crassa* | 13 regulation of or interaction with cellular environment |
| An14g00710 | strong similarity to bud emergence mediator BEM1 - *Saccharomyces cerevisiae* | 14 cell fate |
| An12g00220 | weak similarity to putative NADH dehydrogenase chain precursor - *Homo sapiens* | 40 sub-cellular localisation |
| An12g08090 | weak similarity to actin filament-binding protein b-nexilin - *Rattus norvegicus* | 40 sub-cellular localisation |
| An01e00180 | trnaKctt | tRNA |
| An01e02580 | trnaMcat | tRNA |
| An01e09990 | trnaSaga | tRNA |
| An01e11980 | trnaYgta | tRNA |
| An01e12930 | trnaSaga | tRNA |
| An02e09400 | Trnakctt | tRNA |
| An02e09410 | trnaKttt | tRNA |
| An05e01950 | trnaYgta | tRNA |
| An07e02340 | trnaCgca | tRNA |
| An15e07410 | trnaYgta | tRNA |
| An15e07420 | trnaYgta | tRNA |
| An15e07430 | trnaYgta | tRNA |
| An15e07440 | trnaYgta | tRNA |
